# Canadian Hospitals

**Published:** 1894-11-17

**Authors:** 


### CANADIAN HOSPITALS.

By Our Own Correspondent,

### A VISIT TO THE HOSPITALS OF MONTREAL.—III.

(*Continued from page* 67.)

It would be unfair in me to say much regarding the City Hospital at Montreal, for the very week after my visit its demolition and reconstruction was to be begun. The present building dates from 1821, and certainly the sooner it is replaced by a more modern one the better. It is, however, only fair to say that this condemnation applies only to the medical side of the hospital. A new surgical pavilion, connected with the main building only by a temporary corridor, is as bright and sanitary-looking as one could desire, and the management of the institution seems to be thoughtful and vigorous. Therefore we may hope that with the aid of the half-million dollars (£104,166) collected for the rebuilding, the Montreal General Hospital will be worthy of the place it must hold in the city. Being now the only hospital really within the town boundaries, it will always receive a large proportion of accidents and surgical cases, besides any cases of sickness arriving at the port in ships. That it should be worthy of its work, and a credit to the largest city in Canada, is a matter of the first importance.

As I have said, the surgical pavilion, by which alone I prefer to judge the institution, is bright and attractive. Great use is made of glass for table-tops, cupboards, lockers, and the like, an arrangement which tends not only to a bright appearance, but to a tasteful as well as tidy arrangement of bandages and dressings. The plate-glass chest of antiseptic bandages, arranged in alternate pink and white layers had a distinctly decorative effect. On the other hand I missed the lavish use of the flowers that make the London hospitals so attractive.

There are now 52 nurses in training at the General Hospital. The period of training is two years for probationers between the ages of 25 and 35; any younger ones accepted—and the matron, Miss Livingstone, has not yet found it possible to make the age limit altogether exact—are expected to remain for three years. Nurses begin to receive payment after two months' probation, and then earn six dollars (25s.) a month. During the second year of their training they receive seven, and in the third, eight dollars a month. It is common in many American hospitals to have a male attendant, an orderly, in the male wards. This is an undoubted convenience to the nurses, and has on the face of it much to recommend it, but Miss Livingstone, reflecting that her nurses would not often have such aid at command in private work, concluded that it was better that they should not become used to it in their training. The orderly has therefore been dispensed with. Private patients are received at the rate of two dollars a day. If a special nurse is required two dollars a day more is charged. On these terms the private patient becomes a remunerative inmate of the hospital, though sometimes, in return, a very exacting one.

The gem of all Canadian hospitals is, however, the Royal Victoria Hospital of Montreal. It consists of two handsome pavilions of grey sandstone, united by an administrative building, and is constructed on the best English model, after designs by Mr. Saxon Snell. The original plan has not yet been fully completed, and it is proposed to erect shortly a pathological building at a cost of £8,000.

The Victoria Hospital sprang from a desire on the part of two prominent citizens of Montreal, Sir Donald Smith and Mr. Stephen, now Lord Mountstephen, to erect some lasting memorial of the Queen's Jubilee. Each of these gentlemen contributed half a million dollars for the erection of the hospital. The city gave a site of 13 acres, which, however, was not found suitable, and the founders thereupon bought an adjoining piece of ground at a cost of 86,000 dollars. On this the hospital is built, while the site offered by the city forms the lawns around it. It is estimated that when finished and fully equipped the Victoria Hospital will have cost between eight and nine million dollars, or considerably over a million and a half sterling. The hospital is intended to accommodate 260 patients, but the wards at present open will hold only 152, and the average occupancy since the date of opening on January 2nd of this year has been 146. There are rooms for 22 private patients, and these rooms are not only comfortably but luxuriously furnished. Everything in them is not only new, but of good quality and in the best taste. Indeed, one can, without much strain on the imagination, think of a patient complaining of the plainness of his home surroundings when he leaves the hospital. These patients pay two dollars a day, and are nursed by the staff, but the doctors are allowed to charge extra for their services. As the privilege is confined to the doctors attached to the hospital, there is some jealousy felt by outside practitioners. So much is to be said on both sides of this question, that it is doubtful how it will be finally settled.

An important requirement of perfect freedom from infection is fulfilled by having the patients' clothes invariably sterilised at a temperature of 260 deg. Fahr. The laundry is separate from the main building, and there is also a disinfecting chamber, modelled on that at St. George's Hospital in London. Another thing to be noted is the medical amphitheatre for demonstrations in medical cases. This obviates the discomfort to lecturer, students, and patient of a crowd found the bed, and the often imperfect opportunity for seeing and noting characteristics which ward demonstrations supply. On the other hand, a very admirable feature of the General Hospital is the separate theatre for gynæcological operations, though doubtless many people would consider that where the one theatre is kept perfectly there is no need for another.

In an institution so lately opened the organisation must be to a certain extent tentative. Let the chief officials be as experienced as they may, those who carry out their wishes have generally to learn the value of complete accuracy and prompt obedience. It takes time, under the best hands, to form a routine, a tradition for any hospital. Miss Draper, the matron of the Victoria Hospital, is now training fourteen probationers, and not till these children of her own care are ready will she feel that the nursing is all she could desire. Of her present staff of head nurses, two come from Johns Hopkins Hospital, Baltimore; one from the Illinois Free Hospital, one from Brooklyn City Hospital, one from Toronto General Hospital, and one from St. John's, New Brunswick. Thus each brings a more or less different tradition, which it will take some time to harmonise into the new responsibilities of their surroundings.

